# Screening and evaluation of the strong endogenous promoters in *Pichia pastoris*

**DOI:** 10.1186/s12934-021-01648-6

**Published:** 2021-08-09

**Authors:** Weiwang Dou, Quanchao Zhu, Meihua Zhang, Zuyuan Jia, Wenjun Guan

**Affiliations:** 1 Institute of Pharmaceutical Biotechnology and The Children’s Hospital, Zhejiang University School of Medicine, Hangzhou, 310058 China; 2 Department of Pharmacy, The First Affiliated Hospital, Zhejiang University, Hangzhou, 310003 China

**Keywords:** *Pichia pastoris*, Endogenous promoter, RNA-seq, β-Galactosidase, Biosynthetic pathway

## Abstract

**Background:**

Due to its ability to perform fast and high-density fermentation, *Pichia pastoris* is not only used as an excellent host for heterologous protein expression but also exhibits good potential for efficient biosynthesis of small-molecule compounds. However, basic research on *P. pastoris* lags far behind *Saccharomyces cerevisiae*, resulting in a lack of available biological elements. Especially, fewer strong endogenous promoter elements available for foreign protein expression or construction of biosynthetic pathways were carefully evaluated in *P. pastoris*. Thus, it will be necessary to identify more available endogenous promoters from *P. pastoris*.

**Results:**

Based on RNA-seq and LacZ reporter system, eight strong endogenous promoters contributing to higher transcriptional expression levels and β-galactosidase activities in three frequently-used media were screened out. Among them, the transcriptional expression level contributed by P_0019_, P_0107_, P_0230_, P_0392_, or P_0785_ was basically unchanged during the logarithmic phase and stationary phase of growth. And the transcriptional level contributed by P_0208_ or P_0627_ exhibited a growth-dependent characteristic (a lower expression level during the logarithmic phase and a higher expression level during the stationary phase). After 60 h growth, the β-galactosidase activity contributed by P_0208_, P_0627_, P_0019_, P_0407_, P_0392_, P_0230_, P_0785_, or P_0107_ was relatively lower than P_GAP_ but higher than P_ACT1_. To evaluate the availability of these promoters, several of them were randomly applied to a heterogenous β-carotene biosynthetic pathway in *P. pastoris*, and the highest yield of β-carotene from these mutants was up to 1.07 mg/g. In addition, simultaneously using the same promoter multiple times could result in a notable competitive effect, which might significantly lower the transcriptional expression level of the target gene.

**Conclusions:**

The novel strong endogenous promoter identified in this study adds to the number of promoter elements available in *P. pastoris*. And the competitive effect observed here suggests that a careful pre-evaluation is needed when simultaneously and multiply using the same promoter in one yeast strain. This work also provides an effective strategy to identify more novel biological elements for engineering applications in *P. pastoris*.

**Supplementary Information:**

The online version contains supplementary material available at 10.1186/s12934-021-01648-6.

## Background

As one of the typical methylotrophic yeasts, *Pichia pastoris* (also known as *Komagataella phaffii*) is not only widely used as an excellent host for heterologous protein expression [1] but also exhibits good potential for efficient biosynthesis of economic compounds such as nootkatone [2], carotenoids [3, 4], xanthophylls [5], ricinoleic acid [6], hyaluronic acid [7] and dammarenediol II [8]. Although a lot of work had been carried out in *P. pastoris*, compared with *Saccharomyces cerevisiae*, fewer strong endogenous promoter elements available for foreign protein expression or construction of biosynthetic pathways were carefully investigated in *P. pastoris* [9].

Using methanol as a carbon source is one of the most favorable properties of *P. pastoris*. Several promoters from the methanol utilization (MUT) pathway were identified as the methanol-dependent strong promoters [10], such as P_AOX1_ (the promoter of alcohol oxidase 1) [11], P_FLD1_ (the promoter of formaldehyde dehydrogenase 1) [12], and P_DAS_ (the promoter of dihydroxyacetone synthase) [13]. On the contrary, P_PEX8_ (the promoter of peroxisomal matrix protein) [14] or P_AOX2_ (the promoter of alcohol oxidase 2) [15] was classified as a weak promoter. Considering that these promoters are tightly regulated by methanol, making them useful for the efficient production of foreign proteins when using methanol as a carbon source and inducer [16].

P_GAP_ is the most frequently used constitutive promoter that drives the expression of glyceraldehyde-3-phosphate dehydrogenase at a high level in the medium with glucose or glycerol but at a moderate level in the medium with methanol as a carbon source. Although in many cases, P_AOX1_ works more efficiently than P_GAP_ for heterologous protein production [17, 18]. Some cases also gave the opposite results. Waterham HR et al. proved the expression level of β-lactamase contributed by P_GAP_ in the medium containing glucose was even higher than P_AOX1_ in the medium containing methanol [19]. Similar result was obtained with the P_GAP_ regulated mammalian peptide transporters rPEPT2 by Doring F et al. [20]. A promoter library of mutated P_GAP_ was generated, and it spanned an activity range between 0.6% and 19.6-fold of the wild-type P_GAP_ activity [21]. As another well-known constitutive and strong promoter, the transcriptional activity of P_TEF1_, which controls the expression of translation elongation factor 1 alpha [22], was also found to be similar to or higher than that of P_GAP_ in batch cultures or fed-batch cultures (glucose or glycerol was used as carbon source). P_GCW14_ is a strong constitutive promoter discovered recently [23]. It exhibited stronger promoter activity than P_TEF1_ or P_GAP_ under different carbon sources (glucose, glycerol, or methanol) when an enhanced green fluorescent protein (EGFP) was used as the reporter. Moreover, some other constitutive promoters, P_ENO1_, P_GPM1_, P_HSP82_, P_KAR2_, P_PGK1_, P_SSA4_, and P_TPI1_ [24], were also reported with little attention paid. The expression levels of EGFP contributed by these promoters were showed to be 10–80% of that of P_GAP_.

Although most studies have focused on strong promoters, it is worth noting that strong promoters are not suitable in some applications. For example, promoters with moderate expression levels are sometimes more desirable for transporter or chaperone expression. Thus, the studies on various promoters with different transcriptional activities should also be emphasized. In this study, based on the RNA-seq data and LacZ reporter system, eight novel endogenous promoters that exhibited strong transcriptional activities in three frequently-used media were screened out. A heterogenous β-carotene biosynthetic pathway was chosen to further evaluate the availability of these promoters. It was also found that simultaneously using the same promoter multiple times could result in a notable competitive effect, which might significantly lower the transcriptional expression level of the target gene.

## Results

### Screening the potential strong promoters independent of carbon sources based on RNA-seq

**Fig. 1:**
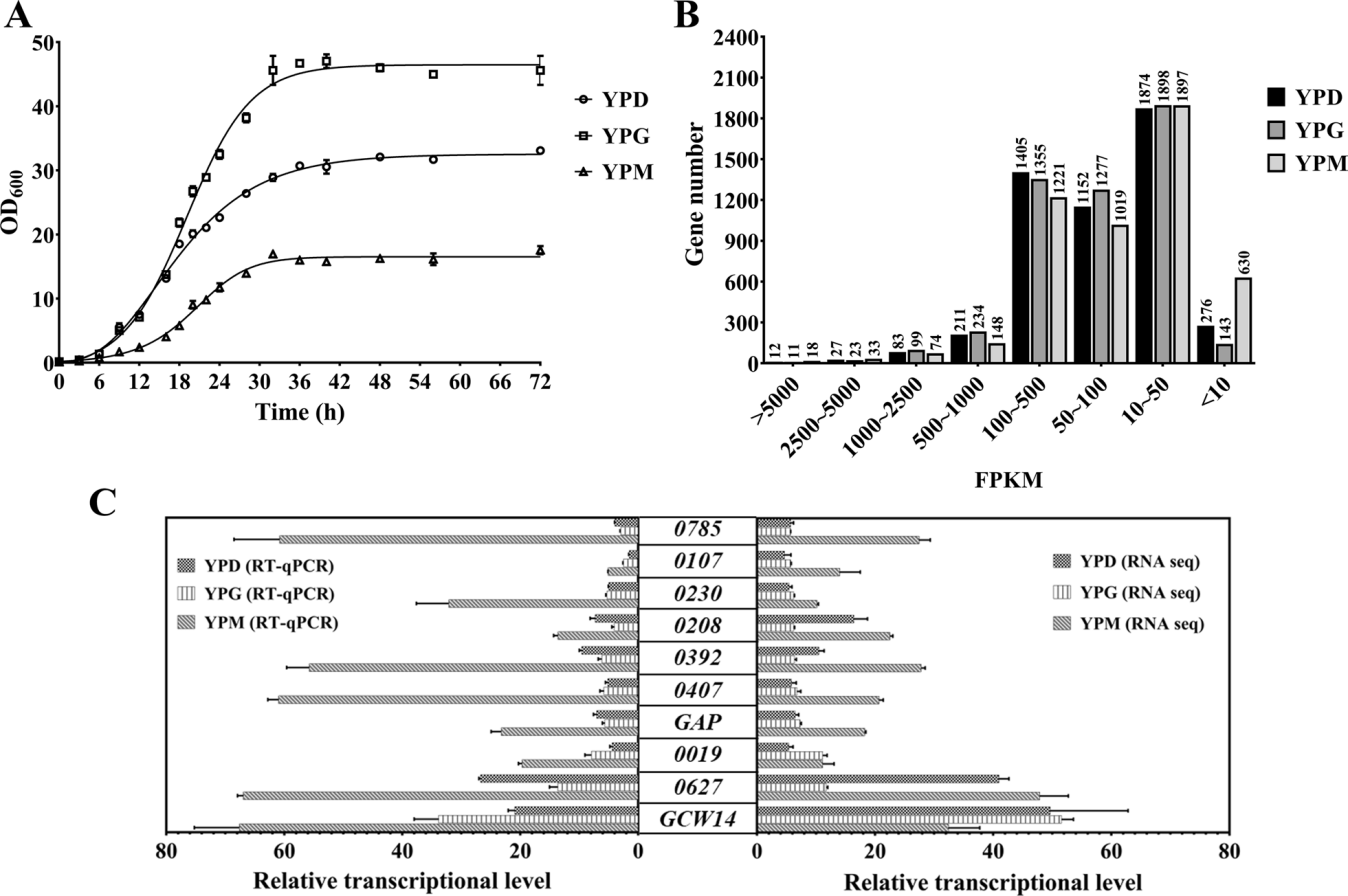
Screening of the strong endogenous promoters based on RNA-seq. **A** The growth curves of *P. pastoris* GS115 WT strain in YPD, YPG, or YPM broth for 72 h with an initial OD_600_ of 0.15. **B** The distribution of FPKM values of all expressed genes in three broths. **C** The RNA-seq and RT-qPCR data of candidate genes at 36 h. Error bars indicate the SD for samples tested in triplicate

In order to screen out strong promoter candidates from *P. pastoris*, the assessment of cell growth status was firstly carried out. As shown in Fig. 1A, the yeast cells gradually entered the stationary phase after 36 h growth in YPD, YPG, or YPM broth. Considering that the accumulation of protein expression or small-molecule compound has occurred chiefly during the stationary phase of yeast growth, total RNA isolation and subsequent RNA-seq were performed after 36 h growth.

As shown in Fig. 1B, the distribution of FPKM (referred to fragments per kilobase of transcript per million base pairs sequenced) values of genes was similar when the GS115 WT strain was cultured in three broths, and the FPKM values of most genes fall in the range of 10–50. The FPKM value of *ACT1* (*PAS_chr3_1169*), which is usually used as an endogenous reference gene (housekeeping gene) in yeast [25, 26], was 624 in YPD, 858 in YPG, and 315 in YPM, respectively. It ranked 245th out of 4764 (YPD), 185th out of 4897 (YPG), and 473th out of 4410 (YPM) expressed genes (FPKM < 10 was defined as not expressed in the corresponding medium), indicating that P_ACT1_ could be considered as a strong promoter. To explore the potential strong promoters in all the above media, the genes with FPKM values five times higher than that of *ACT1* were selected as the candidate genes. Finally, the promoters of *PAS_chr1-4_0586* (*GCW14*), *PAS_chr4_0627, PAS_chr2-2_0019, PAS_chr2-1_0437* (*GAP*), *PAS_chr1-1_0407, PAS_chr2-2_0392, PAS_chr3_0230, PAS_chr2-2_0208, PAS_chr4_0785*, and *PAS_chr1-1_0107* were screened out (Additional file 1). For the subsequent description’s convenience, the above genes were represented by *GCW14*, *0627*, *0019*, *GAP*, *0407*, *0392*, *0230*, *0208*, *0785*, and *0107*, respectively. In addition, the expression levels of these candidate genes were also confirmed by real-time quantitative PCR (RT-qPCR). As shown in Fig. 1C, all the RT-qPCR results were consistent with the RNA-seq data, suggesting these promoters could be used for further evaluation.

### Comparing the transcriptional activities of promoter candidates

**Fig. 2:**
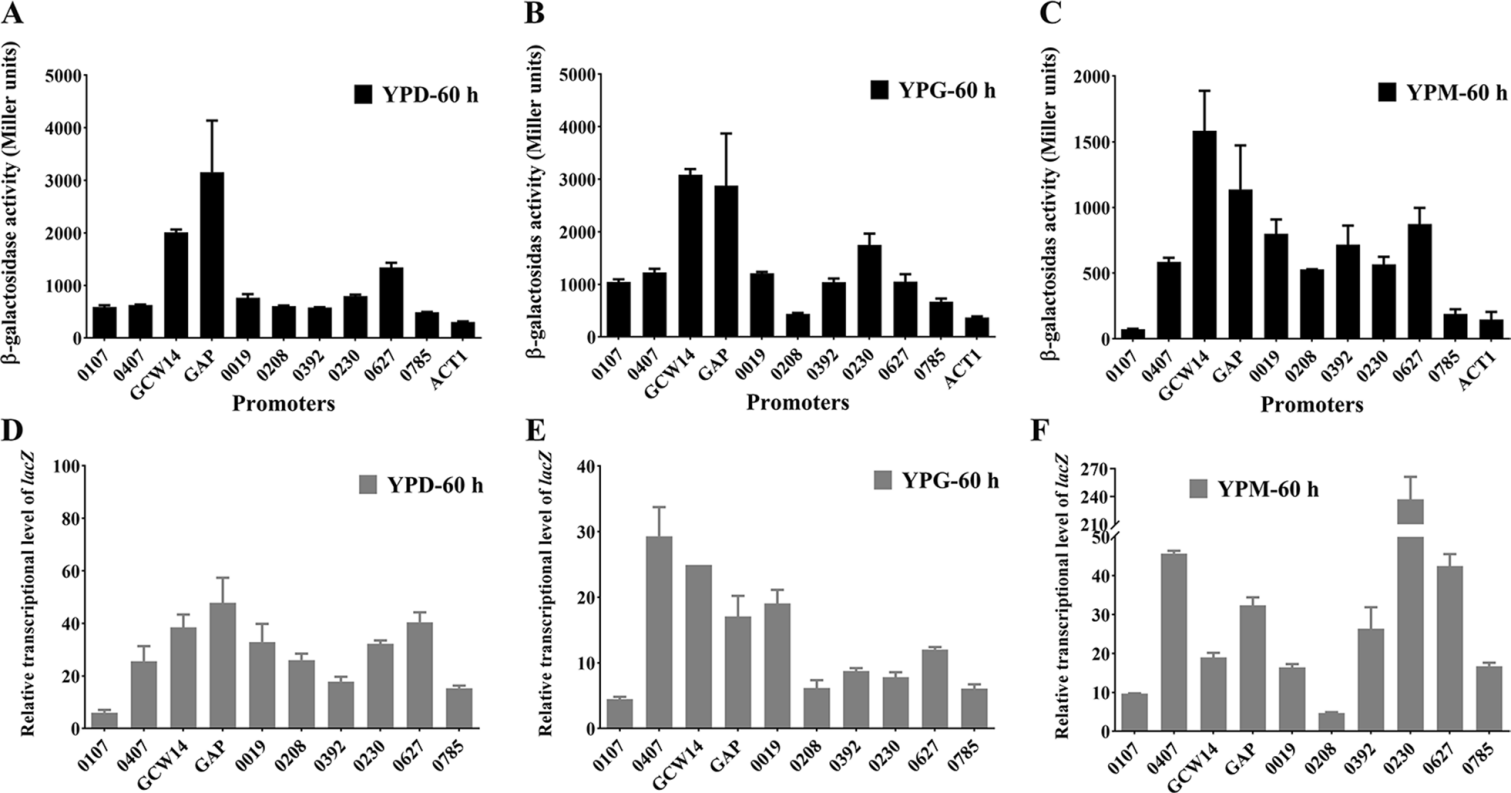
Comparison of the selected promoter activities. **A**–**C** The β-galactosidase activity of transformant harboring vector pZeocin/P_XXX_-*lacZ*-T_ADH1_ (P_XXX_ indicates the name of candidate promoter) after 60 h growth. **D**–**F** The relative transcriptional levels of *lacZ* in different transformants. The *ACT1* gene was used as the reference gene. Error bars indicate the SD for samples tested in triplicate

In order to compare the transcriptional activities of above-selected promoters, the β-galactosidase activities of transformants harboring vector pZeocin/P_XXX_-*lacZ*-T_ADH1_ (P_XXX_ indicates the name of candidate promoter) were measured after 60 h growth. The transcriptional levels of *lacZ* in all transformants were also tested by RT-qPCR. All experimental data was obtained from three independently screened colonies. As shown in Fig. 2, the β-galactosidase activity contributed by each selected promoter was higher than that by P_ACT1_ when the transformant was cultured in three mentioned broths, except P_0107_ which exhibited a lower β-galactosidase activity compared to P_ACT1_ in YPM broth. In YPD or YPG broth, the activity of P_GAP_ or P_GCW14_ was significantly higher than those of the rest promoters. In YPM broth, P_GCW14_ contributed the highest β-galactosidase activity among the all-selected promoters, and P_GAP_, P_0208_, P_0019_, P_0392_, P_0230_, or P_0627_ contributed a similar β-galactosidase activity. In summary, among the ten selected promoters, the previously reported P_GAP_ and P_GCW14_ showed strong transcriptional activities. The transcriptional activity of P_0019_, P_0107_, P_0208_, P_0230_, P_0392_, P_0407_, P_0627_, or P_0785_ was between that of strong promoters P_GAP_ and P_ACT1_. It indicates that these promoter candidates could be recognized as strong endogenous promoters in *P. pastoris*.

Although the transcriptional activities of most selected promoters corresponded with the β-galactosidase activities at 60 h, there were still few exceptions, such as P_0407_ and P_0230_. For example, the transcriptional level of P_0407_-*lacZ* was much higher than that of P_GAP_-*lacZ* when the relative transformants were cultured in YPG or YPM broth. However, the β-galactosidase activities controlled by P_0407_ were less than half of that by P_GAP_. So was P_0230_-*lacZ*. We evaluated the copy number of *lacZ* controlled by each promoter, the results proved all clones harboring only one *lacZ*, eliminating the influence of transcriptional level by copy number (data not shown). It indicated that the transcriptional and protein level were not coordinated in some genes. The reason was not clear.

### P_0208_ and P_0627_ are novel growth-dependent promoters

To evaluate whether the above promoters are constitutive, the β-galactosidase activities of different transformants at 16 h (exponential phase) and 60 h (stationary phase) were measured. In view of P_GAP_ being identified as a recognized constitutive promoter, it was chosen as a control in this study. As shown in Table 1, most promoter candidates, except P_0407_, P_0627_, and P_0208_, could be defined as constitutive promoters due to their relatively stable β-galactosidase activities at 16 and 60 h. The relative β-galactosidase activity driven by P_0208_, P_0407_, or P_0627_ at 16 h was at least 16.5, 2.5 or 7.5 times lower than that at 60 h in three media, reminding us that these promoters might be associated with cell growth.

**Table 1:** The relative β-galactosidase activities driven by the selected promoters compared with P_GAP_

Promoters	YPD 16 h (%)	YPD 60 h (%)	YPG 16 h (%)	YPG 60 h (%)	YPM 16 h (%)	YPM 60 h (%)
P_GAP_	100.00	100.00	100.00	100.00	100.00	100.00
P_ACT1_	17.48	11.25	18.78	15.77	30.43	15.48
P_GCW14_	88.74	74.51	131.99	132.30	162.67	165.46
P_0019_	27.51	28.25	36.94	51.98	88.92	83.48
P_0107_	30.57	21.95	32.84	44.90	6.76	7.59
P_0230_	30.15	29.49	42.96	75.11	54.56	59.04
P_0392_	13.39	21.47	23.55	44.67	59.76	74.83
P_0785_	19.09	18.23	19.52	28.66	34.18	19.71
P_0208_	0.53	22.52	1.14	18.76	0.60	55.25
P_0407_	7.92	23.23	19.13	52.61	8.26	61.18
P_0627_	6.29	49.65	5.99	45.05	7.87	91.24

To test this possibility, the transcriptional levels of *0407*, *0208*, and *0627* genes in the GS115 WT strain were detected at the different growth stages. As shown in Additional file 2, the transcriptional levels of *0407*, *0627* and *0208* genes increased along with the growth time of GS115 WT strain in YPD and YPG media, indicating P_0407_, P_0208_ and P_0627_ could be considered as growth-dependent promoters. In YPM broth, they behaved not growth-dependent at 60 h. We suspect that it may be related to the depletion of carbon source (methanol). In our study, YPD (2%), YPG (2%) or YPM (1%) has 32.58, 33.36 and 12.26 mmol carbon atoms, respectively. At 60 h, the methanol had been depleted which may affect the cell metabolism, so the relative transcriptional level of *0627*, *0407* and *0208* at 60 h was lower than that at 36 h, showing non-growth-dependent characteristic. From 16 to 36 h, the relative transcriptional level was increased. Throughout the performance of *0208* and *0627* in three media for three time points, it demonstrates that they are growth-dependent. In addition, the transcriptional level of *0407* was higher than that of *ACT1* at 16 h in YPD, YPG and YPM (Additional file 2), indicating P_0407_ was not a strictly growth-dependent promoter.

**Fig. 3:**
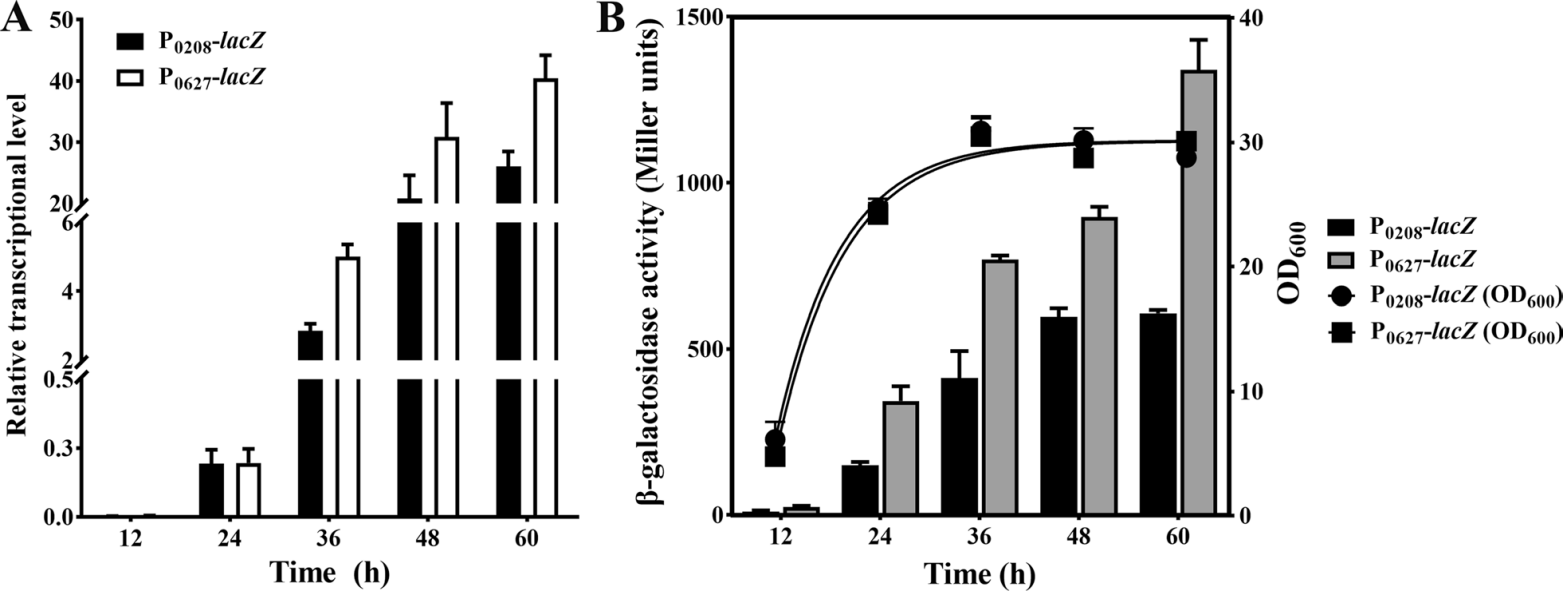
The properties of P_0208_ and P_0627_. **A** The relative transcriptional level of P_0208_-*lacZ* or P_0627_-*lacZ* after 12–60 h growth in YPD broth. The *ACT1* gene was used as the reference gene. **B** The growth curve and β-galactosidase activity of GS115/P_0208_-*lacZ*-T_ADH1_ or GS115/P_0627_-*lacZ*-T_ADH1_ strain cultured in YPD broth. Error bars indicate the SD for samples tested in triplicate

To further investigate the transcriptional properties of P_0208_ and P_0627_, the GS115/P_0208_-*lacZ*-T_ADH1_ and GS115/P_0627_-*lacZ*-T_ADH1_ strains were cultured in YPD broth and sampled every 12 h from 12 h to 60 h. It was found that the relative transcriptional level of P_0208_ was increased from 0.002 to 16 h to 26 at 60 h, while that of P_0627_ was increased from 0.005 to 16 h to 40 at 60 h (Fig. 3A). Although the growth status of GS115/P_0208_-*lacZ*-T_ADH1_ or GS115/P_0627_-*lacZ*-T_ADH1_ strain was similar, the β-galactosidase activity of GS115/P_0208_-*lacZ*-T_ADH1_ strain was lower than that of GS115/P_0627_-*lacZ*-T_ADH1_ strain at each sampling point (Fig. 3B), suggesting that P_0627_ has good potential to be used in expressing the toxic proteins independent of methanol induction.

### Evaluation of the promoter candidates through the heterogenous β-carotene biosynthetic pathway

**Fig. 4:**
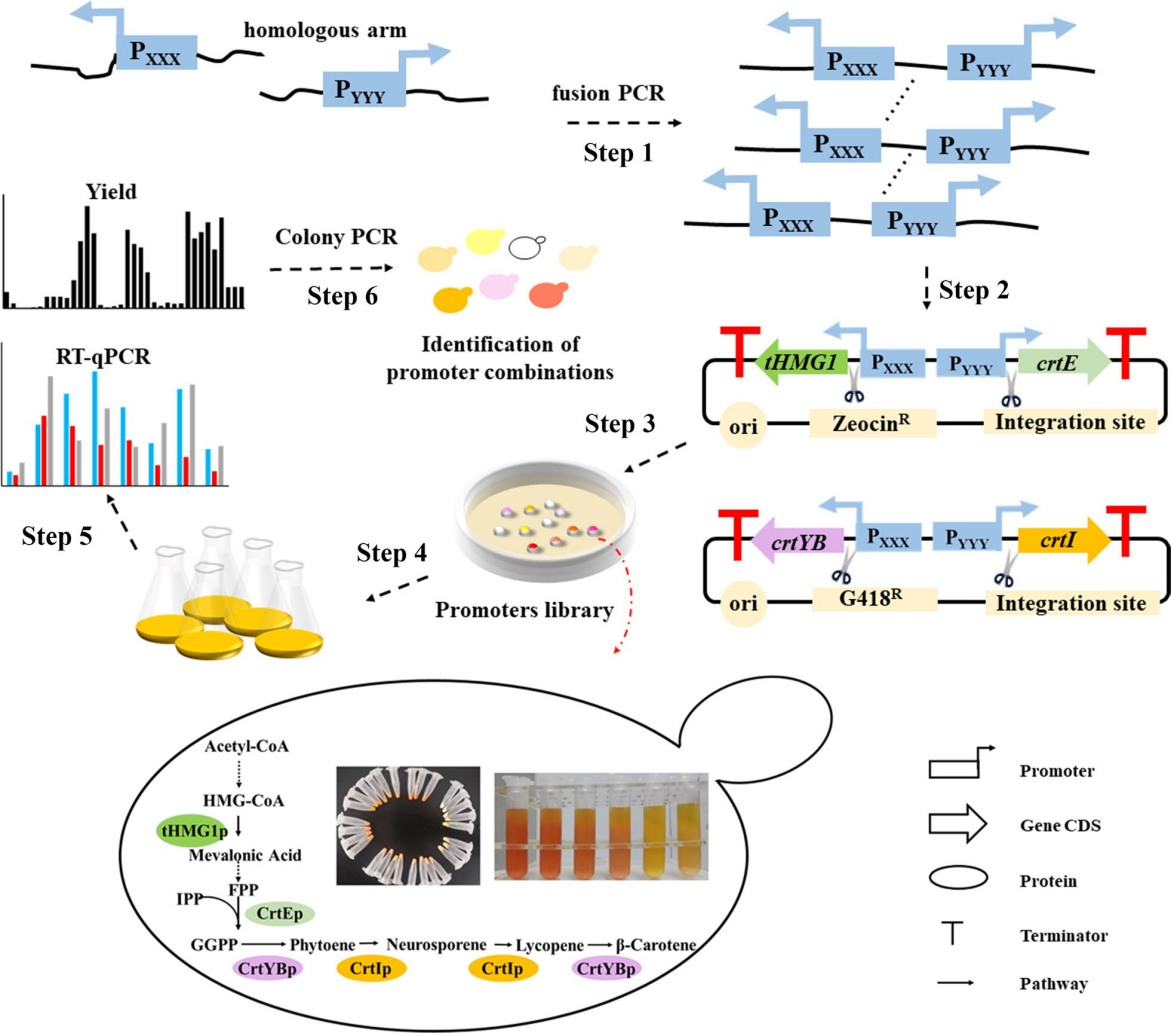
Construction of the β-carotene engineering strain

According to the previous report, the optimized β-carotene biosynthetic pathway always includes four genes, *crtE/tHMG1/crtYB/crtI* [27]. In this study, six promoter candidates (P_GAP_, P_GCW14_, P_0208,_ P_0019_, P_0230_, and P_0107_) with different transcriptional activities were randomly selected and applied to the β-carotene biosynthetic pathway to further evaluate the availability of promoters. As shown in Fig. 4, a promoter library was first constructed by random linkage of two reverse-aligned promoters (Step 1). Then two vectors (pZeocin/P_XXX_*-crtE-*P_YYY_*-tHMG1* and pG418/P_XXX_*-crtYB-*P_YYY_-*crtI*, P_XXX_ and P_YYY_ indicate the name of candidate promoters) containing the above four genes were constructed (Step 2) and transformed into the GS115 WT strain in turn (Step 3). The promoter activities were qualitatively observed by the color of the transformants on plates (Step 4) and then quantitively determined by liquid fermentation (Step 5).

**Fig. 5:**
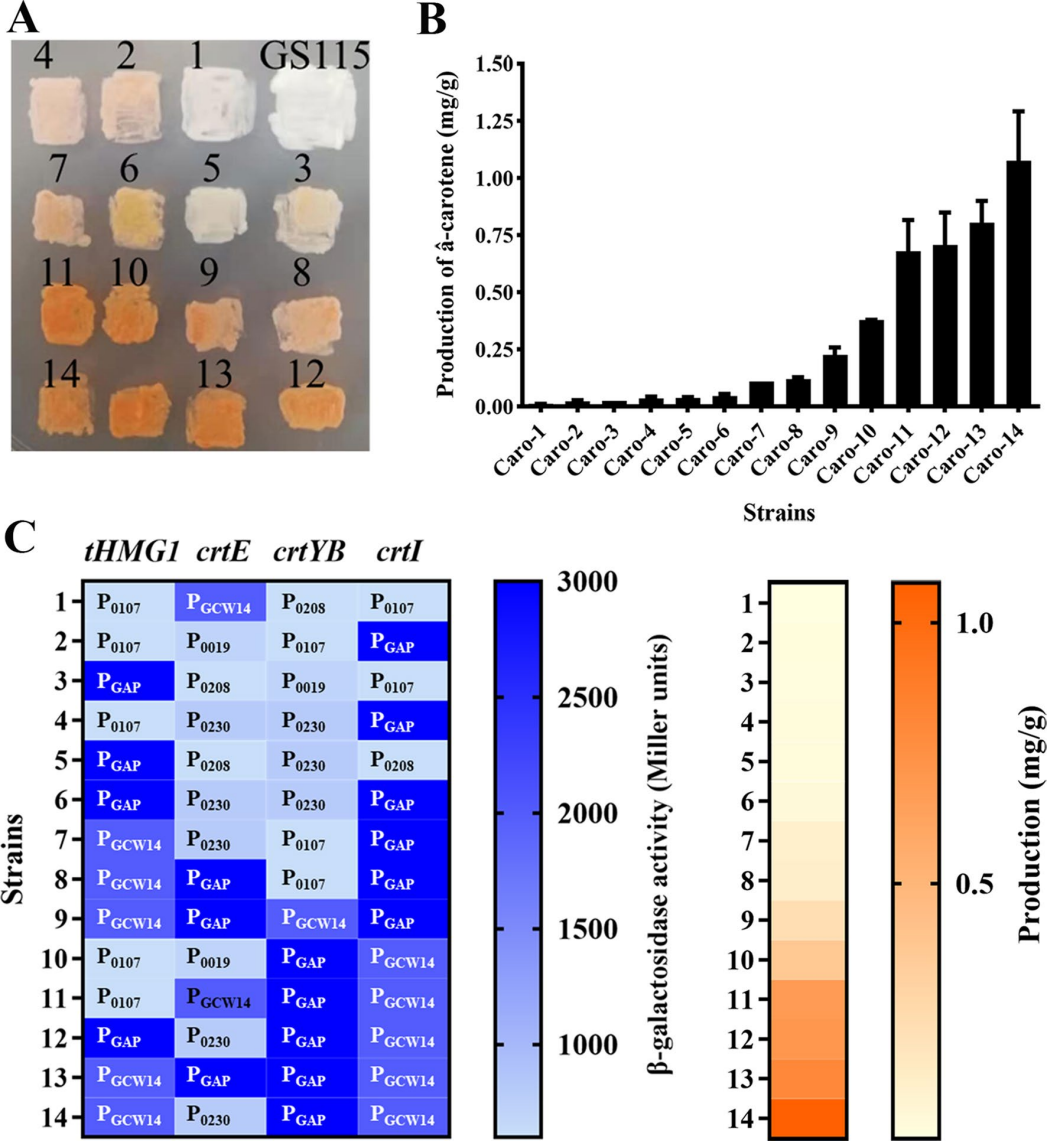
Evaluation of the promoter candidates through the β-carotene biosynthetic pathway. **A** The fourteen engineering strains with significant color differences grown on the YPD agar plate. **B** The production of β-carotene of the selected engineering strains. Error bars indicate the SD for samples tested in triplicate. **C** Heat map of the transcriptional levels (blue) and the β-carotene productions (orange)

Finally, fourteen transformants with significant color differences were screened out for further liquid fermentation (Fig. 5A). The result indicates that the darker the color of transformant, the higher the yield of β-carotene (Fig. 5B). And the yield of β-carotene from these yeast mutants was from 0.0123 mg/g DCW (Caro-1 strain) up to 1.07 mg/g DCW (Caro-14 strain).

The promoters of four genes (*crtE, tHMG1*, *crtYB*, and *crtI*) in each strain were verified by PCR and the primers were shown in Additional file 3. The relationship between the transcriptional activity of each gene and the β-carotene production of the engineering strain was then analyzed. As shown in Fig. 5C, when both *crtYB* and *crtI* had a stronger promoter (similar to P_GAP_), the β-carotene production of engineered strain was generally higher, and when *crtYB* had a relatively weaker promoter compared to P_GAP_, the β-carotene production of engineered strain was decreased. This result was consistent with the previous report that CrtYBp was the rate-limiting enzyme in β-carotene production [28]. Compared to the promoter combinations in the β-carotene biosynthetic pathway of Caro-13 and Caro-14 strain, all corresponding genes used the same promoter except *crtE.* The promoter of *crtE* was P_GAP_ in the Caro-13 strain and P_0230_ in the Caro-14 strain. Although the transcriptional activity of P_0230_ was lower than that of P_GAP_ at 60 h, the β-carotene production of the Caro-14 strain was much higher than that of the Caro-13 strain. This result suggested that not all genes should have stronger transcriptional levels to achieve higher β-carotene production. Balancing the expression level of each gene in the biosynthetic pathway could be a better choice.

### The existence of a significant competitive effect when one promoter was used multiple times in a yeast strain

**Fig. 6:**
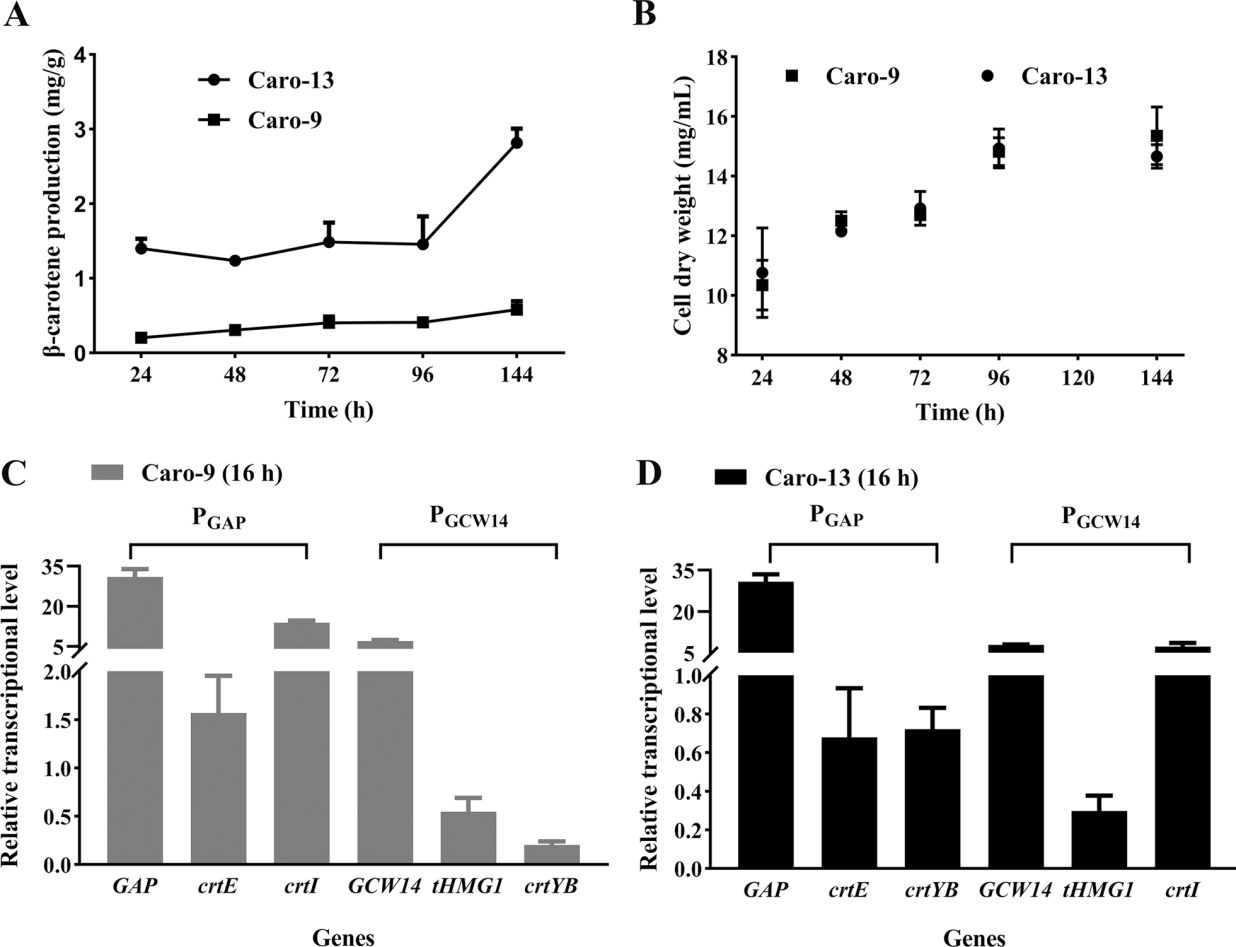
The competitive effect existing in Caro-13 and Caro-9 strains. **A** A significant difference in the production of β-carotene in both strains. **B** The growth curves of both strains. **C** The relative transcriptional levels of the exogenous genes in the Caro-9 strain after 16 h growth. **D** The relative transcriptional levels of the exogenous genes in the Caro-13 strain after 16 h growth. The *ACT1* gene was used as the reference gene. Error bars indicate the SD for samples tested in triplicate

In the Caro-9 strain, the combination of exogenous genes and promoters was P_GCW14_-*tHMG1*/P_GAP_-*crtE/*P_GCW14_-*crtYB/*P_GAP_-*crtI*. In the Caro-13 strain, that was P_GCW14_-*tHMG1*/P_GAP_-*crtE*/P_GCW14_-*crtI/*P_GAP_-*crtYB*. Although both P_GAP_ and P_GCW14_ were strong promoters, and their related β-galactosidase activities were similar (Table 1), the β-carotene production of Caro-9 strain was over 3-fold lower than that of Caro-13 strain (Fig. 6A, B). The RT-qPCR result at 16 h also showed that the transcriptional level of *crtYB* in the Caro-13 strain was over 3-fold higher than that in the Caro-9 strain (Fig. 6C). To figure out the reason, the transcriptional levels of four genes in the Caro-9 or Caro-13 strain were measured, respectively. Accidentally, the transcriptional levels of two exogenous genes controlled by the same promoter behaved differently. As shown in Fig. 6C, although an endogenous gene (*GAP*) and two exogenous genes (*crtE* and *crtI*) were all driven by P_GAP_ in the Caro-9 strain, the transcriptional levels of both exogenous genes were much lower than that of *GAP*, presenting a significant “competitive effect”. In addition, the transcriptional levels of *crtE* and *crtI* were also different. So were *GCW14, crtYB*, and *tHMG1*controlled by P_GCW14_. A similar phenomenon was also observed in the Caro-13 strain (Fig. 6D). Such a “competitive effect” when the same promoter was used multiple times in one yeast strain resulted in a lower expression level of the related gene than expected. It might be an important factor limiting the β-carotene yields of engineering strains constructed in this study.

## Discussion

The strong promoter is an indispensable element in the engineering retrofit and application of yeast. Due to the lack of basic research compared with *S. cerevisiae*, the availability of strong promoter is scarce in *P. pastoris*, which seriously restricts the subsequent application.

In this study, by analyzing the RNA-seq data of GS115 WT strain in the stationary phase under different media (YPD, YPG, and YPM), ten strong endogenous promoters (P_GCW14_, P_0627_, P_0019_, P_GAP_, P_0407_, P_0392_, P_0230_, P_0208_, P_0785_, and P_0107_) which were not affected by carbon sources were screened out. Except for P_GCW14_ and P_GAP_, eight of them were first reported here. Five (P_0019_, P_0392_, P_0230_, P_0785_, and P_0107_) were identified as strong constitutive promoters, and two (P_0627_ and P_0208_) as growth-dependent strong promoters. The growth-dependent transcriptional behavior of P_0627_ or P_0208_ makes it a good selection for the expression of toxic foreign proteins in *P. pastoris*. P_0407_ showed a similar trend as P_0627_ and P_0208_ in β-galactosidase activity. However, the transcriptional level of P_0407_-*lacZ* was higher than that of *ACT1* at 16 h, indicating this promoter was not a strictly growth-dependent promoter. In addition, in our study, the activity of P_GCW14_ was lower than that of P_GAP_ in YPD broth and similar to that of P_GAP_ in YPG broth when measured with the β-galactosidase activity assay. These results were not consistent with the previous report that P_GCW14_ activity was much higher than P_GAP_ by EGFP reporter in YPD or YPD broth [23].

However, the results of RT-qPCR and the β-galactosidase activity did not agree in all cases. For example, the transcriptional level of P_0407_-*lacZ* was much higher than that of P_GAP_-*lacZ* when the relative transformants were cultured in YPG or YPM broth. However, its related β-galactosidase activity was much lower than P_GAP_. P_0107_ presented the lowest transcriptional level at 60 h, but its corresponding β-galactosidase activity was not the lowest (Fig. 2). These results agree with the suggestion of previous reports that it was not that the higher of transcriptional level, the higher of protein expression level [29, 30].

In this study, a heterogenous biosynthetic pathway of β-carotene containing four genes was used to further evaluate the properties of six strong endogenous promoters (P_GAP_, P_GCW14_, P_0208_, P_0019_, P_0230_, and P_0107_). The Caro-14 strain contributed the highest yield of β-carotene (1.07 mg/g DCW). It is higher than the yield in *S. cerevisiae* containing integrated carotenogenic gene cassettes *crtYB/I/E/tHMG1* (501 µg/g DCW) [31], suggesting that *P. pastoris* might have the advantages to be engineered as a carotene producer.

In addition, the competitive effect was first evaluated in this study. It was found that using the same promoter multiple times in one strain could lead to a significant decrease of the expression level of the target gene, reminding a careful evaluation is required before using a promoter in this way.

Some reports considered a 500 bp upstream sequence covering the whole promoter region of most genes in *S. cerevisiae* [32, 33]. As the most known strong constitutive promoter P_GAP_ in *P. pastoris*, its’ sequence length is 477 bp. It should be noted that the intergenic region between *GAP* and its neighbor gene was 1576 bp. On the contrary, the sequence length of another well-known strong inducible promoter, P_AOX1_, is 939 bp in the commercial vector pPICZαC, mainly covering the intergenic region between *AOX1* and its neighbor gene (998 bp). The study of P_AOX1_ showed that the region − 1055 to − 809 and − 653 to − 514 significantly affects the promoter activity [34]. In our study, the 500 bp upstream sequence of candidate gene was amplified to act as a promoter. However, the lengths of the intergenic region of some genes are more than 500 bp, which might cause incomplete promoter elements like P_AOX1_, thereby affecting their normal functions. The corresponding intergenic regions of six selected promoters are over 500 bp except P_0208_, reminding the possibility that several key regulatory elements might not be included in the 500 bp sequence (Additional file 4). The promoter length would be appropriately extended in the follow-up study to explore whether it will significantly impact promoter activity.

## Method and materials

### Strains, vectors, and media

All strains used in this study are listed in Additional file 5. *Escherichia coli* TG1 was used to amplify plasmids in LB medium (0.5% yeast extract, 1% tryptone, 1% sodium chloride) containing 100 µg/mL ampicillin, zeocin, or 50 µg/mL kanamycin sulfate, and incubated at 37 °C. The yeast mutants were derived from the *P. pastoris* GS115 wild-type strain. All yeast strains, unless otherwise specified, were grown in YPD medium (1% yeast extract, 2% peptone, 2% glucose) at 30 °C.

### Measurement of the yeast growth curves in different carbon source broths

The GS115 WT strain was pre-cultured in 5 mL YPD broth at 30 °C, 220 rpm for 24 h and followed by inoculating to an initial OD_600_ of 0.1 in 250 mL shaking flasks containing 50 mL YPD (2% glucose), YPG (2% glycerol), or YPM (1% methanol) for 72 h. The optical absorbance (OD_600_) of the culture was detected by an Evolution™ 220 spectrophotometer (Thermo Fisher Scientific Inc., USA), and each medium had three replicates.

### RNA-seq analysis

Three independent clones of the GS115 WT strain were incubated in 5 mL YPD broth for 24 h. The pre-cultured pellet was washed with presterilized water and transferred into YPD, YPG, or YPM broth for incubating. The yeast cells (total OD_600_ of 16) were harvested by centrifugation (5000 *g* for 5 min, 4 °C) at 36 h when they entered the stationary phase. Total RNA was isolated using a Yeast RNA kit (Omega Bio-Tek Inc., USA) through the operation manual. The residuary genomic DNA was eliminated using a DNase I treatment (Takara Bio Inc., Japan). The qualified RNA samples determined with Bioanalyzer 2100 (Agilent Technologies, USA) were used for library construction with the sequencing platform BGISEQ-500 (BGI Shenzhen, China). Compared to the genome, the average comparison rate of the sample is 97.13%, and the average comparison rate of the compared gene set is 82.58%. All the generated raw sequencing reads were filtered to remove reads with adaptors, reads with more than 10% unknown bases, and low-quality reads. Clean reads were then obtained and stored in FASTQ format. The clean reads were mapped to the genome of GS115 by HISAT [35]. The software package RSEM [36] was used to quantify the gene expression level. The ratio of FPKM for candidate genes to that for *ACT1* was used as the relative fold change for RNA-seq data treatment.

### Real-time quantitative PCR (RT-qPCR) analysis

The methods for obtaining pure total RNA were mentioned above. After DNase I treatment, the absence of DNA contamination was confirmed by PCR using a pair of genomic primers, and the genome of the GS115 WT strain was used as a positive control. 800 ng of total RNA was used to reverse transcription to generate cDNA with HiScript II  Reverse Transcriptase (Vazyme Biotech Co., Ltd., China). RT-qPCR was carried out on a Mastercycler® ep gradient S instrument (Eppendorf, Hamburg, Germany) with 2 × ChemQ SYBR qPCR Mester Mix (Vazyme Biotech Co., Ltd., China). The *ACT1* gene was chosen as an internal control to normalize the relative expression levels of target genes. All reactions were performed in three replicates. A ΔΔCt method was used for the relative fold change analysis [37].

### Construction of the vectors containing P_XXX_-*lacZ* and yeast transformation

The genomic DNA of *E. coli* BL21 (DE3) was used as a template to amplify the open reading frame (ORF) of *lacZ.* DNA fragments of the integration site (*AOX1* promoter, which helped the plasmid inserted into the chromosome) were amplified from GS115. *ADH1* terminator (T_ADH1_) was amplified from the genomic DNA of *S. cerevisiae* BY4741. The fragment of ori and zeocin-resistant marker came from the commercial plasmid pPICZαC (Invitrogen Corporation, USA). All the fragments were amplified with Hieff Canace™ high-fidelity DNA Polymerase (Yeasen, China) and then be linked by the homologous arm with pEASY®-Basic Seamless Cloning and Assembly Kit (TransGen Biotech Co., Ltd, China) to construct a vector pZeocin/*lacZ*-T_ADH1_.

The promoter fragments of candidates were amplified from the genomic DNA of *P. pastoris* GS115 by PCR and then inserted into the *HindIII* site of pZeocin/*lacZ*-T_ADH1_ with seamless cloning to yield vector pZeocin/P_XXX_-*lacZ*-T_ADH1_ (P_XXX_ indicates the name of candidate promoters).

The linearized vector pZeocin/P_XXX_-*lacZ*-T_ADH1_ with *PmeI* (located in the middle of *AOX1* promoter locus) digested were transferred into the GS115 cells by electro-transformation and integrated into the genome by homologous recombination [38]. The strains containing P_XXX_-*lacZ* were selected from the agar plates containing zeocin (0.1 mg/mL) and verified by PCR. The template came from colony or genomic DNA. The colony was boiled in water for 5 min before PCR and the genomic DNA was extracted as the previous report [39].

### β-Galactosidase activity assay

The β-galactosidase activity was assayed according to the previous report with slight modification [40]. Briefly, the yeast cells (total OD_600_ of 3) were collected. The supernatant was discarded by centrifuge, and the pellet was resuspended in 1 mL Z buffer (60 mM Na_2_HPO_4_, 40 mM NaH_2_PO_4_, 10 mM KCl, and 1 mM MgSO_4_) and diluted to make the final OD_600_ of 0.2–0.6. Take suitable volumes of the above cells into a 2 mL Eppendorf tube, and make up to 1 mL with Z Buffer. Add 25 µL 0.1% SDS and 50 µL chloroform, vortex for 30 s, and incubate at 30 °C for 15 min. The reaction was started through the addition of 200 µL of 4 mg/mL onitrophenyl-β-D-galactopyranoside (ONPG) solution at 30 °C and stopped by the addition of 500 µL of 1 M NaCO_3_. After removal of cell debris by centrifugation, the OD_420_ and OD_550_ of the reaction solution were detected by Evolution™ 220 spectrophotometer, and the relative activity of β-galactosidase was calculated using the formula below:

$${\text{Miller units}} = 1000 \times \left( {{\text{OD}}_{{420}} - 1.75 \times {\text{OD}}_{{550}} } \right)/\left( {{\text{V}} \times {\text{OD}}_{{600}} \times {\text{t}}} \right)$$

T is the reaction time (min) and V is the volume (mL) of culture used for the assay.

### Construction of promoter library in the β-carotene biosynthetic pathway

The vector pMRI-34-*crtE-tHMG1* and vector pMRI-35-*crtYB-crtI* were the kind gifts from Hongwei Yu’s lab [41]. The two gene cassettes with opposite directions of *tHMG1*/*crtE* or *crtI/crtYB* containing the promoter, ORF, and terminator were amplified from the above vectors. The *AOX1* promoter (located in GS115 chromosome 4) and *ENO2* promoter (located in GS115 chromosome 3) were chosen as the integration site. The *ble* (coding zeocin-resistant protein in *E. coli* and yeast) and *kan* (coding kanamycin-resistant protein in *E. coli* and G418-resistant protein in yeast, respectively) were chosen as screening markers. All those fragments were linked with the homologous arm by seamless cloning to yield vector pZeocin/*crtE-tHMG1* and vector pG418/*crtYB-crtI.*

The candidate promoters (P_0107_, P_GCW14_, P_0019_, P_GAP_, P_0208_, and P_0230_) were amplified from the genomic DNA of GS115 by PCR. Two promoters in opposite directions were co-amplified by fusion PCR. Then a pool of combinations with two reversed promoters was obtained. Those promoters were inserted into *BamHI/NotI* sites of vector pZeocin/*crtE-tHMG1* and vector pG418/*crtYB-crtI* with seamless cloning to yield vector pZeocin/P_XXX_*-crtE-*P_YYY_*-tHMG1* and vector pG418/P_XXX_*-crtYB-*P_YYY_*-crtI* (P_XXX_ and P_YYY_ indicate the name of candidate promoters). The vector pZeocin/P_XXX_*-crtE-*P_YYY_*-tHMG1* was linearized with *PmeI* and then transferred into the GS115 cell by electro-transformation. All the transformants grown on the agar plates containing zeocin were selected and co-cultured for the next round of electro-transformation with linearized pG418/P_XXX_*-crtYB-*P_YYY_*-crtI* by *ScaI* in *ENO2* promoter. The transformants with different colors on the agar plates containing G418 (0.25 mg/mL) were picked out. The promoter combinations were verified by PCR.

### Measurement of the β-carotene production

The extraction and analysis of β-carotene were performed according to a previous report [41]. Briefly, the β-carotene engineering strains were pre-cultured in 5 mL YPD broth at 30 °C, 220 rpm for 24 h. Precultures were inoculated to an initial OD_600_ of 0.05 in 20 mL of YPD broth with 100 mL flasks and grown under the same condition for 120 h. Samples were taken every 24 h to determine the DCW and β-carotene yield. The carotenoids were co-extracted using hot HCl-acetone. The analyses of β-carotene were performed on an HPLC system (LC 20AT) equipped with a Supersil ODS2 C18 column (4.6mm × 250mm). The mobile phase is composed of acetonitrile, methanol, and isopropanol with a volume ratio of 5:3:2, followed by a flow rate of 1 mL/min at 40 °C. The UV/VIS signals were detected at 450 nm, and the analysis time of each sample is 40 min. The standard product of β-carotene was purchased from Solarbio (Beijing Solarbio Science & Technology Co., Ltd., China). The standard curve of concentration with the peak area was verified by triplicate.

## Supplementary Information

**Additional file 1.** Identified promoter candidates in *P. pastoris* GS115.

**Additional file 2**. Evaluation of the growth-dependent strong promoter P_0208_ andP_0627_. (A) The relative transcriptional levels of *0627, 0208*,and *0407* in YPD broth (B) The relative transcriptional levels of *0627,0208*, and *0407* in YPG broth (C) The relative transcriptional levelsof *0627, 0208*, and *0407* in YPM broth.

**Additional file 3.** Primers used for RT-qPCR and colony verification.

**Additional file 4.** Sequence length of the intergenic region of selected genes.

**Additional file 5.** Strains and vectors used in this study.

## Data Availability

The datasets used and/or analyzed during the current study are available from the corresponding author on reasonable request.

## Abbreviations

MUT: The methanol utilization
AOX1: Alcohol oxidase 1
FLD1: Formaldehyde dehydrogenase 1
DAS: Dihydroxyacetone synthase
PEX8: Peroxisomal matrix protein
AOX2: Alcohol oxidase 2
FPKM: Fragments per kilobase of transcript per million base pairs sequenced
RT-qPCR: Real‐time quantitative PCR
GPI: Glycosylphosphatidylinositol
GS: Glutamine synthetase
DCW: Dry cell weight
ORF: Open reading frame
T_ADH1_: *ADH1* terminator
ONPG: Onitrophenyl-β-d-galactopyranoside
