# Supplementary material for: Screening and evaluation of the strong endogenous promoters in Pichia pastoris
Source: Microb Cell Fact. 2021 Aug 9;20:156. doi: 10.1186/s12934-021-01648-6 (PMC8351359; doi:10.1186/s12934-021-01648-6)
Supplement: Supplementary file 1 — Additional file 1. Identified promoter candidates in P. pastoris GS115. [file 12934_2021_1648_MOESM1_ESM.docx]

| Gene ID | Gene Name | Annotation |
| --- | --- | --- |
| *PAS_chr1-4_0586* | *GCW14* | A potential glycosyl phosphatidyl inositol (GPI)-anchored protein. |
| *PAS_chr4_0627* | *0627* | Plasma membrane localized protein that protects membranes from desiccation. |
| *PAS_chr2-2_0019* | *0019* | Peroxisomal 2,4-dienoyl-CoA reductase, auxiliary enzyme of fatty acid beta-oxidation. |
| *PAS_chr2-1_0437* | *GAP* | Glyceraldehyde-3-phosphate dehydrogenase, isozyme 3, involved in glycolysis and gluconeogenesis |
| *PAS_chr1-1_0407* | *0407* | Putative protein of unknown function |
| *PAS_chr2-2_0392* | *0392* | Mitochondrial porin (voltage-dependent anion channel), outer membrane protein |
| *PAS_chr3_0230* | *0230* | ATPase involved in protein folding and the response to stress |
| *PAS_chr2-2_0208* | *0208* | Hypothetical protein *PAS_chr2-2_0208* |
| *PAS_chr4_0785* | *0785* | Glutamine synthetase (GS), synthesizes glutamine from glutamate and ammonia |
| *PAS_chr1-1_0107* | *0107* | NADP (+)-dependent glutamate dehydrogenase |
