# Supplementary figures and images for: Screening and evaluation of the strong endogenous promoters in Pichia pastoris
Source: Microb Cell Fact. 2021 Aug 9;20:156. doi: 10.1186/s12934-021-01648-6 (PMC8351359; doi:10.1186/s12934-021-01648-6)

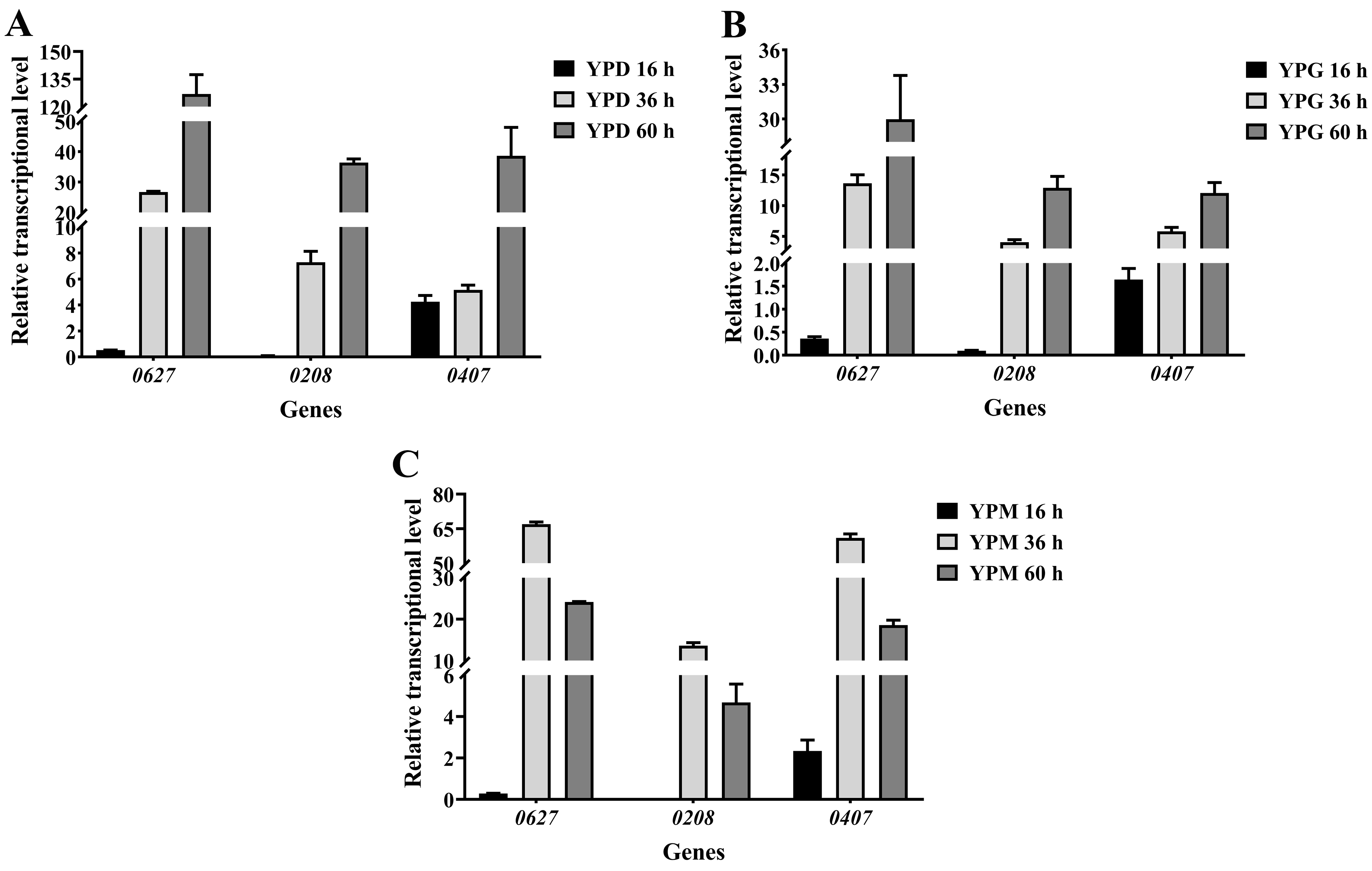

Supplement: Supplementary file 2 — Additional file 2. Evaluation of the growth-dependent strong promoter P0208 andP0627. (A) The relative transcriptional levels of 0627, 0208,and 0407 in YPD broth (B) The relative transcriptional levels of 0627,0208, and 0407 in YPG broth (C) The relative transcriptional levelsof 0627, 0208, and 0407 in YPM broth. [file 12934_2021_1648_MOESM2_ESM.tif]

| Promoters | The length of intergenic region (bp) | Schematic diagram |
| --- | --- | --- |
| PGAP | 1576 | 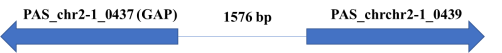 |
| PAOX1 | 998 | 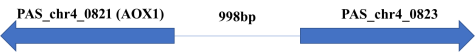 |
| PGCW14 | 857 | 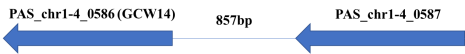 |
| P0019 | 2448 | 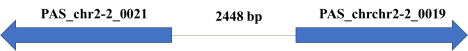 |
| P0107 | 811 | 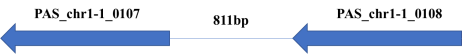 |
| P0230 | 529 | 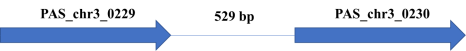 |
| P0392 | 295 | 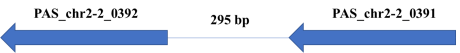 |
| P0785 | 1194 | 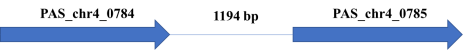 |
| P0208 | 397 | 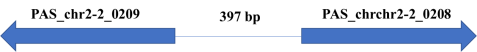 |
| P0407 | 1543 | 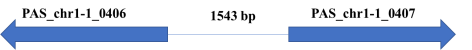 |
| P0627 | 665 | 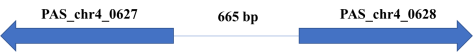 |

Supplement: Supplementary file 4 — Additional file 4. Sequence length of the intergenic region of selected genes. [file 12934_2021_1648_MOESM4_ESM.docx]
