# Supplementary material for: Screening and evaluation of the strong endogenous promoters in Pichia pastoris
Source: Microb Cell Fact. 2021 Aug 9;20:156. doi: 10.1186/s12934-021-01648-6 (PMC8351359; doi:10.1186/s12934-021-01648-6)
Supplement: Supplementary file 3 — Additional file 3. Primers used for RT-qPCR and colony verification. [file 12934_2021_1648_MOESM3_ESM.docx]

| RT-qPCR primers | Sequence (5’-3’) |
| --- | --- |
| PAS_chr1-4_0586 F204 | ACCATCTCCAAGACCACCAAG |
| PAS_chr1-4_0586 R347 | CAGCAACACCAGCCTTAGCAG |
| PAS_chr4_0627 F114 | CAAGGCTGCCAACAAATTGAC |
| PAS_chr4_0627 R250 | TTACCAGCTTCCAATGCATCACC |
| PAS_chr2-2_0208 F183 | TGAACAAGTGGCTCCTCACGA |
| PAS_chr2-2_0208 R323 | TCCCTCCTTCAAATCATTGGCTT |
| PAS_chr2-2_0392 F462 | CATTAATGCCGCTTCCGTCAC |
| PAS_chr2-2_0392 R595 | AGCTTTACCACCTACCTCGAC |
| PAS_chr1-1_0407 F297 | GCATCATCACGTTGACAAACA |
| PAS_chr1-1_0407 R441 | ACTGTCCATTTCCGGTCAAGA |
| PAS_chr4_0785 F143 | AGCCTTCTTCCATTGATGACC |
| PAS_chr4_0785 R278 | AGCACTCGGCCAGAACAACAA |
| PAS_chr3_0230 F1079 | ACAAGTCCATCAACCCAGACG |
| PAS_chr3_0230 R1216 | CTTGGTCATGATACCACCAGC |
| PAS_chr2-2_0019 F589 | GCTTGCGAATGGGCTCCTTAC |
| PAS_chr2-2_0019 R722 | CGTTAGGTTCAGCTAGCCTCT |
| GAP F685 | GTTTGGCTTTCCGTGTCCCA |
| GAP R819 | CGGCATCTTCAGTGTAACCC |
| PAS_chr1-4_0570 F631 | ACCACTTACGGAACCAAGAC |
| PAS_chr1-4_0570 R771 | ACTTCATAGCCTCGTCGACT |
| ACT1 RT F893 | TGCAAAAGGAGCTTACTGCC |
| ACT1 RT R1037 | TGGTCCAGATTCGTCGTACT |
| crtYB RT F3 | GCCTACGCAGAGGATCTTGC |
| crtYB RT R3 | TCCATCCGGCAACTGTCCTT |
| crtI RT F2 | CAAGCGCAACAATCCCTCAG |
| crtI RT R2 | GGAGGCGTAAACGAGGTCAG |
| tHMG1 RT F2 | TCAACTGGATCGAAGGTCGT |
| tHMG1 RT R2 | AAATCCACCAACAGACCCAG |
| crtE RT F2 | TCGGCCTCGTCTGAAAACGG |
| crtE RT R2 | TCTTCGCTAGGACACGTCAG |
| LacZ RT F3 | GCACGGCAGATACACTTGCT |
| LacZ RT R3 | CGGTGTATCGCTCGCCACTT |
| crtI RT 6F | ACGATGCCAGAAACAAGATT |
| crtI RT 6R | AGTCCTCGGCCAAGAAGATA |
|  | |
| colony verification | Sequence (5’-3’) |
| PGAP F2 | GCCCGTTACCGTCCCTAGGA |
| P0019 F2 | GACCCTGGTTGCTACATTTG |
| P0208 F2 | CCATGAAAGGTTCAAAGGCA |
| P0230 F | AGCCGCAGGAACCATATGTG |
| P0107 F | CAATCACAAGCGGAAACACC |
| tHMG1 R | AGCTTTCAATATCGCGGGAA |
| crtE R | CCAACAACGTTCTGGATGAC |
| crtYB R | CCACTCTCCGCTGATGGATA |
| crtI R | GGCGATTCCACCGATACCAC |
