# Supplementary material for: Screening and evaluation of the strong endogenous promoters in Pichia pastoris
Source: Microb Cell Fact. 2021 Aug 9;20:156. doi: 10.1186/s12934-021-01648-6 (PMC8351359; doi:10.1186/s12934-021-01648-6)
Supplement: Supplementary file 5 — Additional file 5. Strains and vectors used in this study. [file 12934_2021_1648_MOESM5_ESM.docx]

| **Strains or vectors** | **Description** | **Source** |
| --- | --- | --- |
| **Stains** | | |
| *E. coli* TG1 | Host strain for DNA clone | Invitrogen |
| *S. cerevisae* BY4741 | Provide genome for fragment amplification | Invitrogen |
| *P. pastoris* GS115 | his4^-^, the basic strain for this study | Invitrogen |
| GS115-P_0107_-lacZ | P_AOX1_ :: P_0107_-*lacZ*-T_ADH1,_ Zeocin^R^ | This study |
| GS115-P_0407_-lacZ | P_AOX1_ :: P_0407_-*lacZ*-T_ADH1,_ Zeocin^R^ | This study |
| GS115-P_0019_-lacZ | P_AOX1_ :: P_0019_-*lacZ*-T_ADH1,_ Zeocin^R^ | This study |
| GS115-P_0208_-lacZ | P_AOX1_ :: P_0208_-*lacZ*-T_ADH1,_ Zeocin^R^ | This study |
| GS115-P_0230_-lacZ | P_AOX1_ :: P_0230_-*lacZ*-T_ADH1,_ Zeocin^R^ | This study |
| GS115-P_0392_-lacZ | P_AOX1_ :: P_0392_-*lacZ*-T_ADH1,_ Zeocin^R^ | This study |
| GS115-P_0785_-lacZ | P_AOX1_ :: P_0785_-*lacZ*-T_ADH1,_ Zeocin^R^ | This study |
| GS115-P_0627_-lacZ | P_AOX1_ :: P_0627_-*lacZ*-T_ADH1,_ Zeocin^R^ | This study |
| GS115-P_GAP_-lacZ | P_AOX1_ :: P_GAP_-*lacZ-*T_ADH1,_ Zeocin^R^ | This study |
| GS115-P_GCW14_-lacZ | P_AOX1_ :: P_GCW14_-*lacZ*-T_ADH1,_ Zeocin^R^ | This study |
| GS115-P_ACT1_-lacZ | P_AOX1_ :: P_ACT1_-*lacZ*-T_ADH1,_ Zeocin^R^ | This study |
| Caro-1 | GS115, P_AOX1_::P_0107_-*tHMG1*-T_GCW14_+  P_GCW14_-*crtE*-T_GAP_, ZeocinR, P_ENO2_::P_0208_-*crtYB*-T_GCW14_+P_0107_-*crtI*-T_GAP_, G418^R^ | This study |
| Caro-2 | GS115, P_AOX1_::P_0107_-*tHMG1*-T_GCW14_+  P_0019_-*crtE*-T_GAP_, ZeocinR, P_ENO2_::P_0107_-*crtYB*-T_GCW14_+P_GAP_-*crtI*-T_GAP_, G418^R^ | This study |
| Caro-3 | GS115, P_AOX1_::P_GAP_-*tHMG1*-T_GCW14_+  P_0208_-*crtE*-T_GAP_, ZeocinR, P_ENO2_::P_0019_-*crtYB*-T_GCW14_+P_0107_-*crtI*-T_GAP_, G418^R^ | This study |
| Caro-4 | GS115, P_AOX1_::P_0107_-*tHMG1*-T_GCW14_+  P_0230_-*crtE*-T_GAP_, ZeocinR, P_ENO2_::P_0230_-*crtYB*-T_GCW14_+P_GAP_-*crtI*-T_GAP_, G418^R^ | This study |
| Caro-5 | GS115, P_AOX1_::P_GAP_-*tHMG1*-T_GCW14_+  P_0208_-*crtE*-T_GAP_, ZeocinR, P_ENO2_::P_0230_-*crtYB*-T_GCW14_+P_0208_-*crtI*-T_GAP_, G418^R^ | This study |
| Caro-6 | GS115, P_AOX1_::P_GAP_-*tHMG1*-T_GCW14_+  P_0230_-*crtE*-T_GAP_, ZeocinR, P_ENO2_::P_0230_-*crtYB*-T_GCW14_+P_GAP_-*crtI*-T_GAP_, G418^R^ | This study |
| Caro-7 | GS115, P_AOX1_::P_GCW14_-*tHMG1*-T_GCW14_+  P_0230_-*crtE*-T_GAP_, ZeocinR, P_ENO2_::P_0107_*-crtYB*-T_GCW14_+P_GAP_-*crtI*-T_GAP_, G418^R^ | This study |
| Caro-8 | GS115, P_AOX1_::P_GCW14_-*tHMG1*-T_GCW14_+  P_GAP_-*crtE*-T_GAP_, ZeocinR, P_ENO2_::P_0107_-*crtYB*-T_GCW14_+P_GAP_-*crtI*-T_GAP_, G418^R^ | This study |
| Caro-9 | GS115, P_GCW14_-*tHMG1*-T_GCW14_+  P_GAP_-*crtE-*T_GAP_, ZeocinR, P_ENO2_::P_GCW14_-*crtYB*-T_GCW14_+P_GAP_-*crtI*-T_GAP_, G418^R^ | This study |
| Caro-10 | GS115, P_AOX1_::P_0107_-*tHMG1*-T_GCW14_+  P_0019_-*crtE*-T_GAP_, ZeocinR, P_ENO2_::P_GAP_-*crtYB*-T_GCW14_+P_GCW14_-*crtI*-T_GAP_, G418^R^ | This study |
| Caro-11 | GS115, P_AOX1_::P_0107_-*tHMG1*-T_GCW14_+  P_GCW14_-*crtE*-T_GAP_, ZeocinR, P_ENO2_::P_GAP_-*crtYB*-T_GCW14_+P_GCW14_-*crtI*-T_GAP_, G418^R^ | This study |
| Caro-12 | GS115, P_AOX1_::P_0107_-*tHMG1*-T_GCW14_+  P_GCW14_-*crtE*-T_GAP_, ZeocinR, P_ENO2_::P_GAP_-*crtYB*-T_GCW14_+P_GCW14_-*crtI*-T_GAP_, G418^R^ | This study |
| Caro-13 | GS115, P_AOX1_::P_GCW14_-*tHMG1*-T_GCW14_+  P_GAP_-*crtE*-T_GAP_, ZeocinR, P_ENO2_::P_GAP_-*crtYB*-T_GCW14_+P_GCW14_-*crtI*-T_GAP_, G418^R^ | This study |
| Caro-14 | GS115, P_AOX1_::P_GCW14_-*tHMG1*-T_GCW14_+  P_0230_-*crtE*-T_GAP_, ZeocinR, P_ENO2_::P_GAP_-*crtYB*-T_GCW14_+P_GCW14_-*crtI*-T_GAP_, G418^R^ | This study |
| **Vectors** | | |
| pZeocin/P_xxx_-lacZ-T_ADH1_ | The promoter was one of the selected promoters in this study, the *ADH1* terminator came from *S. cerevisiae.* | This study |
| pPICZαC | Containing P_AOX1_, Multiple cloning sites, and T_AOX1_, Zeocin^R^ | Invitrogen |
| pMRI-34-*crtE-tHMG1* | Containing c*rtE* and *tHMG1* | [1] |
| pMRI-35-*crtYB-crtI* | Containing *crtYB* and *crtI* | [1] |
| pZeocin/P_XXX_*-crtE-*P_YYY_*-tHMG1* | Containing c*rtE* and *tHMG1* with different promoter combinations, Zeocin^R^ Amp^R^ | This study |
| pG418/P_XXX_*-crtYB-*P_YYY_*-crtI* | Containing *crtYB* and *crtI* with different promoter combinations, G418^R^ Amp^R^ | This study |

**Reference**

1. Xie WP, Liu M, Lv XM, Lu WQ, Gu JL, Yu HW: Construction of a controllable beta-carotene biosynthetic pathway by decentralized assembly strategy in *Saccharomyces cerevisiae*. *Biotechnol Bioeng* 2014, 111:125-133.
